# Supplementary material for: Predicting Core-Level X‑ray Photoemission Spectra of Oxide Surfaces from First-Principles—A Case Study for SnO2
Source: J Am Chem Soc. 2026 Oct 9;148(41):44934–43. doi: 10.1021/jacs.6c15126 (PMC13693387; doi:10.1021/jacs.6c15126)
Supplement: Supplementary file 1 [file ja6c15126_si_001.pdf]

Supplementary Information for "Predicting core-level X-ray photoemission spectra of oxide surfaces from first principles - a case study for SnO_2"

Wenxuan Cai,[†] Stefan Kucharski,[‡] Christopher Blackman,[‡] Juhan Matthias Kahk,[¶] and Johannes Lischner[*,†]

†*Department of Materials, Imperial College London, London, SW7 2AZ, UK*

‡*Department of Chemistry, University College London, London, WC1H 0AJ, UK*

¶*Institute of Physics, University of Tartu, Tartu, EST-50411, Estonia*

E-mail: j.lischner@imperial.ac.uk

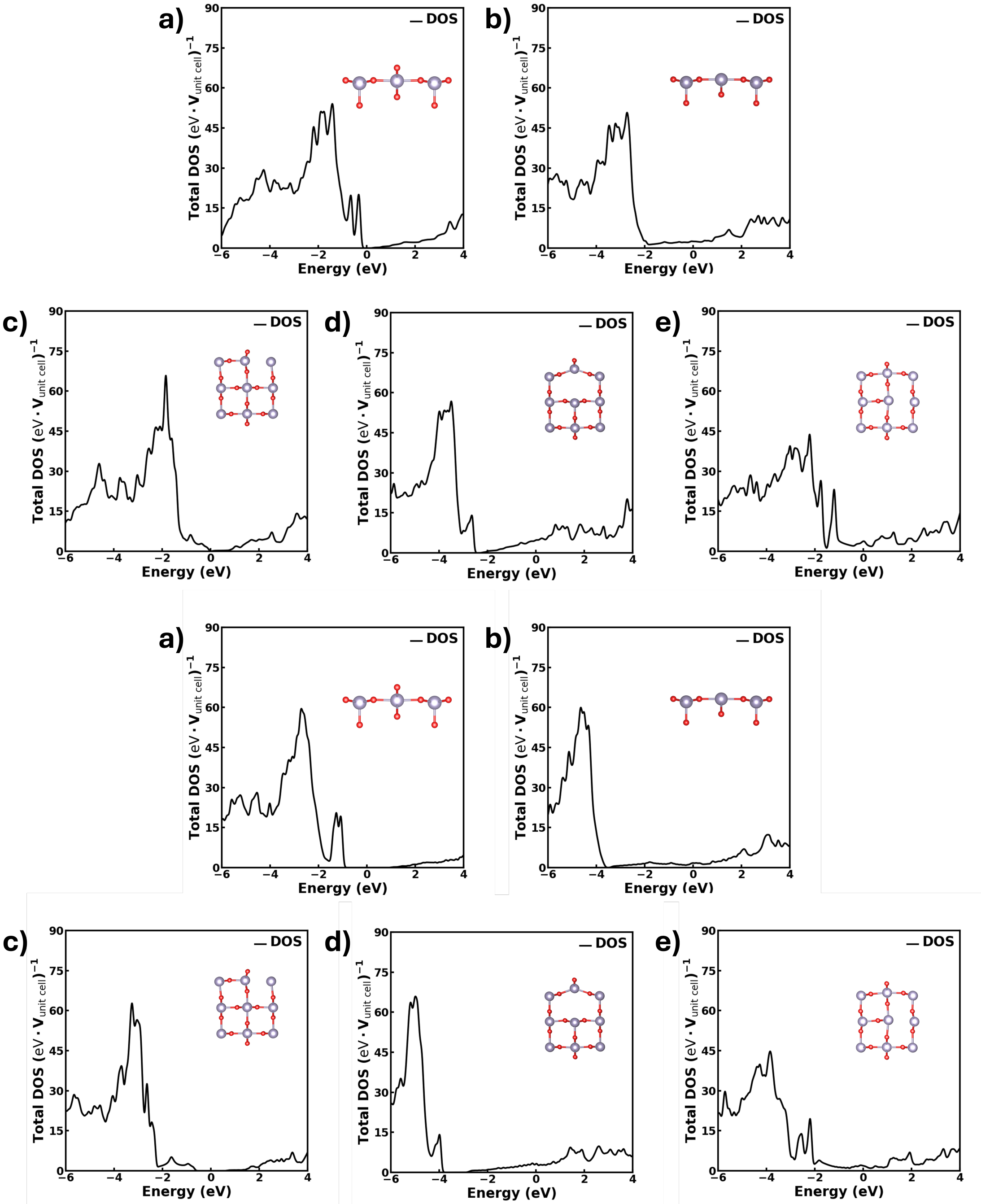

Figure 1: Comparison of densities of states from PBE (top panels) and B3LYP (bottom panels) for different SnO_2 surfaces.

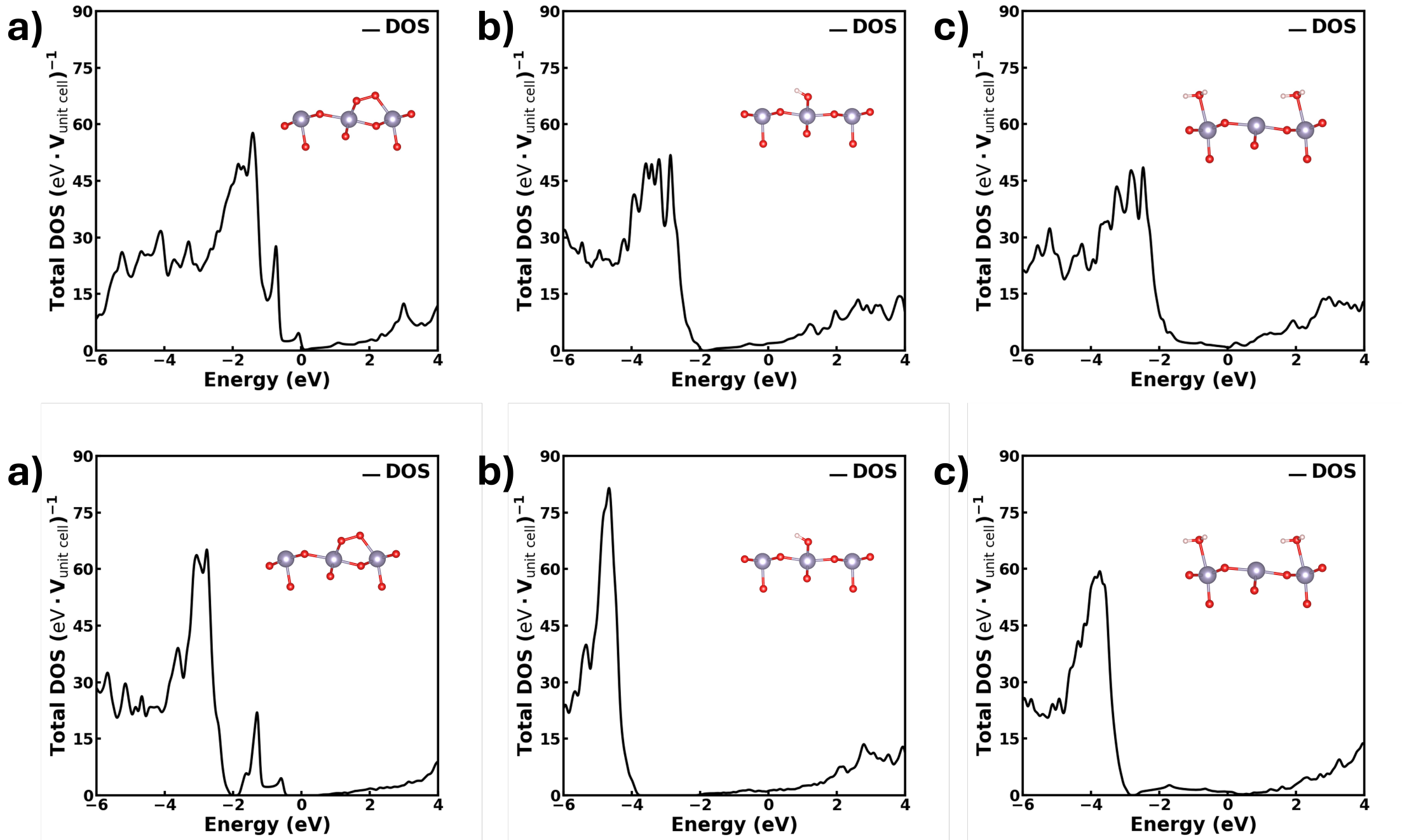

Figure 2: Comparison of densities of states from PBE (top panels) and B3LYP (bottom panels) for different SnO_2 surfaces.

Table 1: Comparison of O 1s CEBE shifts from Δ-SCF and Z+1 methods for bridging oxygen atoms and in-plane oxygen atoms of a fully reduced $SnO_2(110)$ surface. Calculations were performed using 3×3 supercells for both 3-layer and 5-layer slabs. All shifts are referenced to the O atom in the center of the slab.

Site	No. of layers	Δ-SCF shift (eV)	Z+1 shift (eV)	Difference (eV)
In-plane O	3	-0.44	-0.66	0.22
In-plane O	5	-0.40	-0.41	0.01
Sub-bridging O	3	-0.56	-0.65	0.09
Sub-bridging O	5	-0.39	-0.38	-0.01

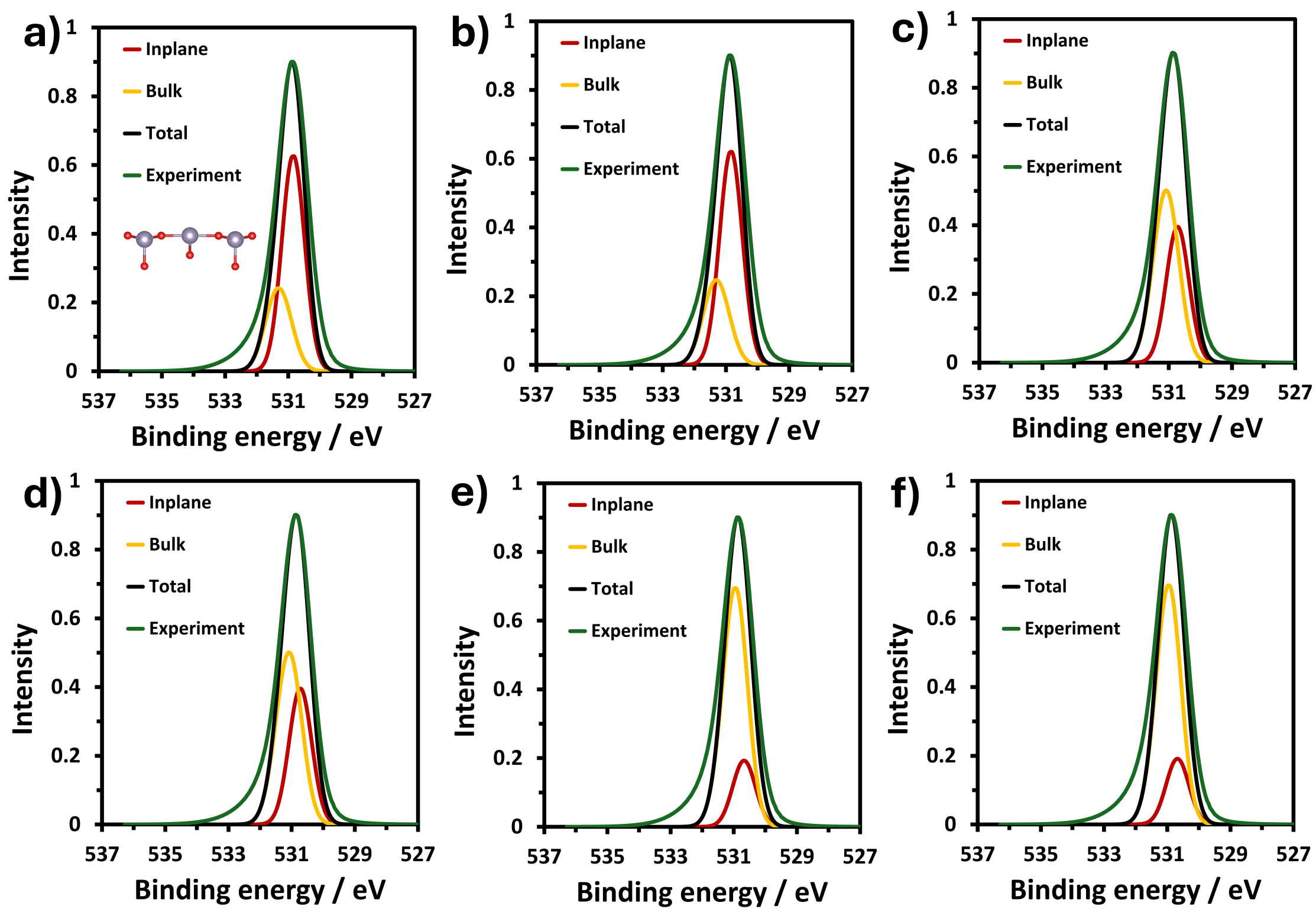

Figure 3: Comparison of simulated and experimental XPS spectra of the fully reduced surface for different values of λ ($\sigma = 0.6$ eV): a) $\lambda = 2.0$ Å, $z = 0$ set for in-plane oxygens, b) $\lambda = 2.0$ Å, $z = 0$ set for bridging oxygens, c) $\lambda = 4.0$ Å, $z = 0$ to in-plane oxygens, d) $\lambda = 4.0$ Å, $z = 0$ set to bridging oxygens, e) $\lambda = 9.0$ Å , $z = 0$ set to in-plane oxygens, f) $\lambda = 9.0$ Å , $z = 0$ set to bridging oxygens.

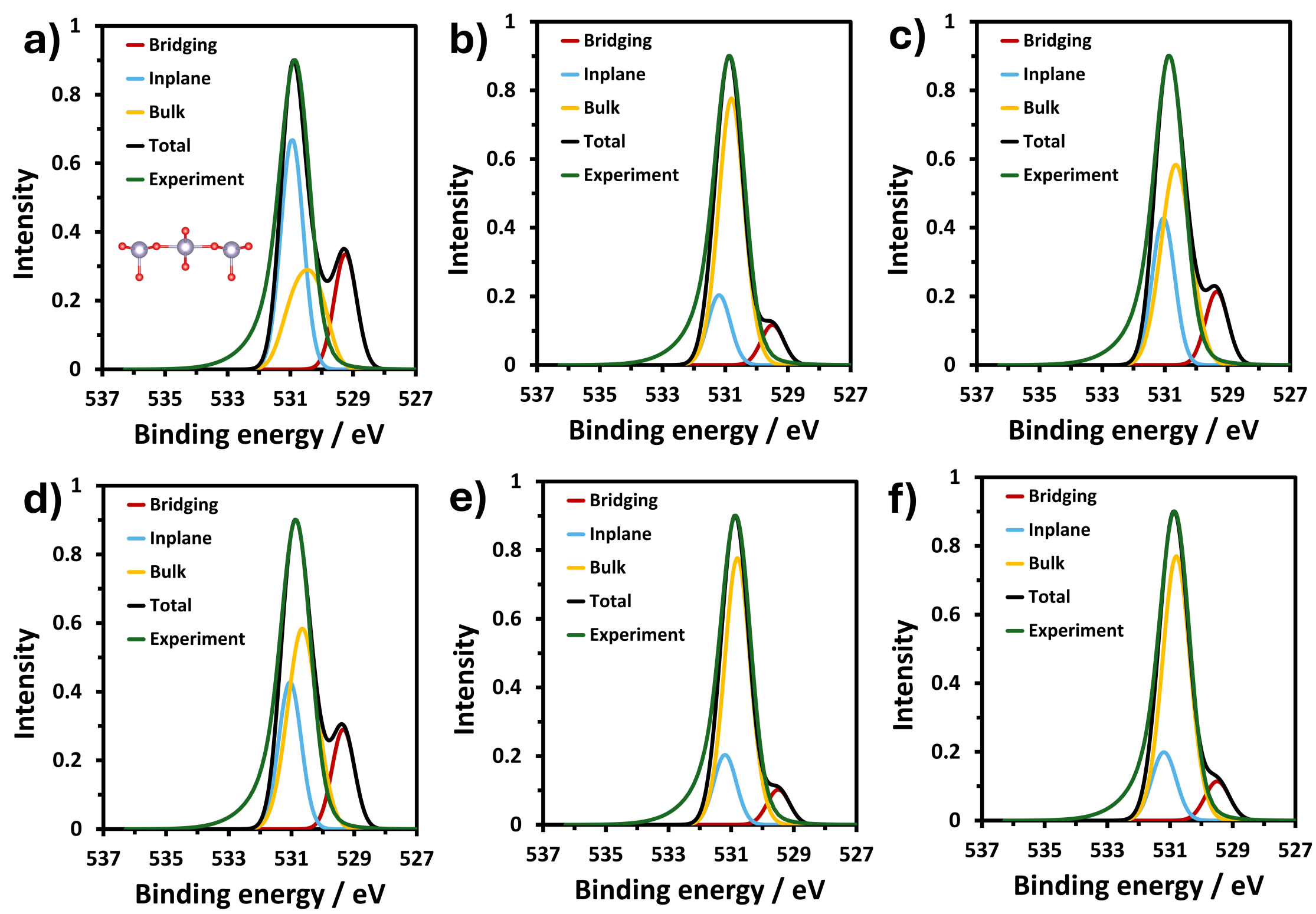

Figure 4: Comparison of simulated and experimental XPS spectra of the stoichiometric surface for different values of λ ($\sigma = 0.6$ eV): a) $\lambda = 2.0$ Å, $z = 0$ set for in-plane oxygens, b) $\lambda = 2.0$ Å, $z = 0$ set for bridging oxygens, c) $\lambda = 4.0$ Å, $z = 0$ to in-plane oxygens, d) $\lambda = 4.0$ Å, $z = 0$ set to bridging oxygens, e) $\lambda = 9.0$ Å , $z = 0$ set to in-plane oxygens, f) $\lambda = 9.0$ Å , $z = 0$ set to bridging oxygens.

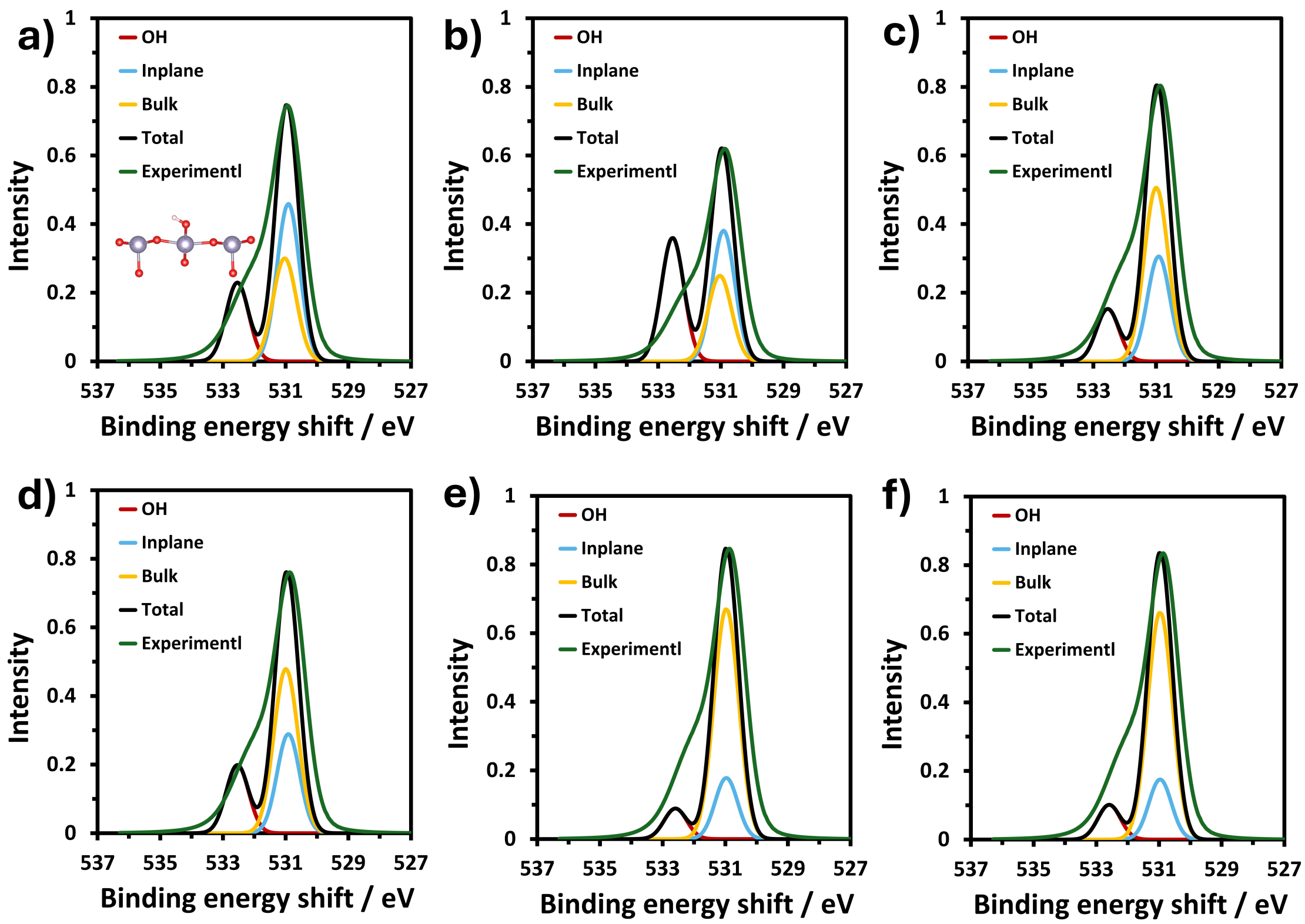

Figure 5: Comparison of simulated and experimental XPS spectra of the fully reduced surface with adsorbed OH for different values of λ ($\sigma = 0.6$ eV): a) $\lambda = 2.0$ Å, $z = 0$ set for in-plane oxygens, b) $\lambda = 2.0$ Å, $z = 0$ set for bridging oxygens, c) $\lambda = 4.0$ Å, $z = 0$ to in-plane oxygens, d) $\lambda = 4.0$ Å, $z = 0$ set to bridging oxygens, e) $\lambda = 9.0$ Å , $z = 0$ set to in-plane oxygens, f) $\lambda = 9.0$ Å , $z = 0$ set to bridging oxygens.

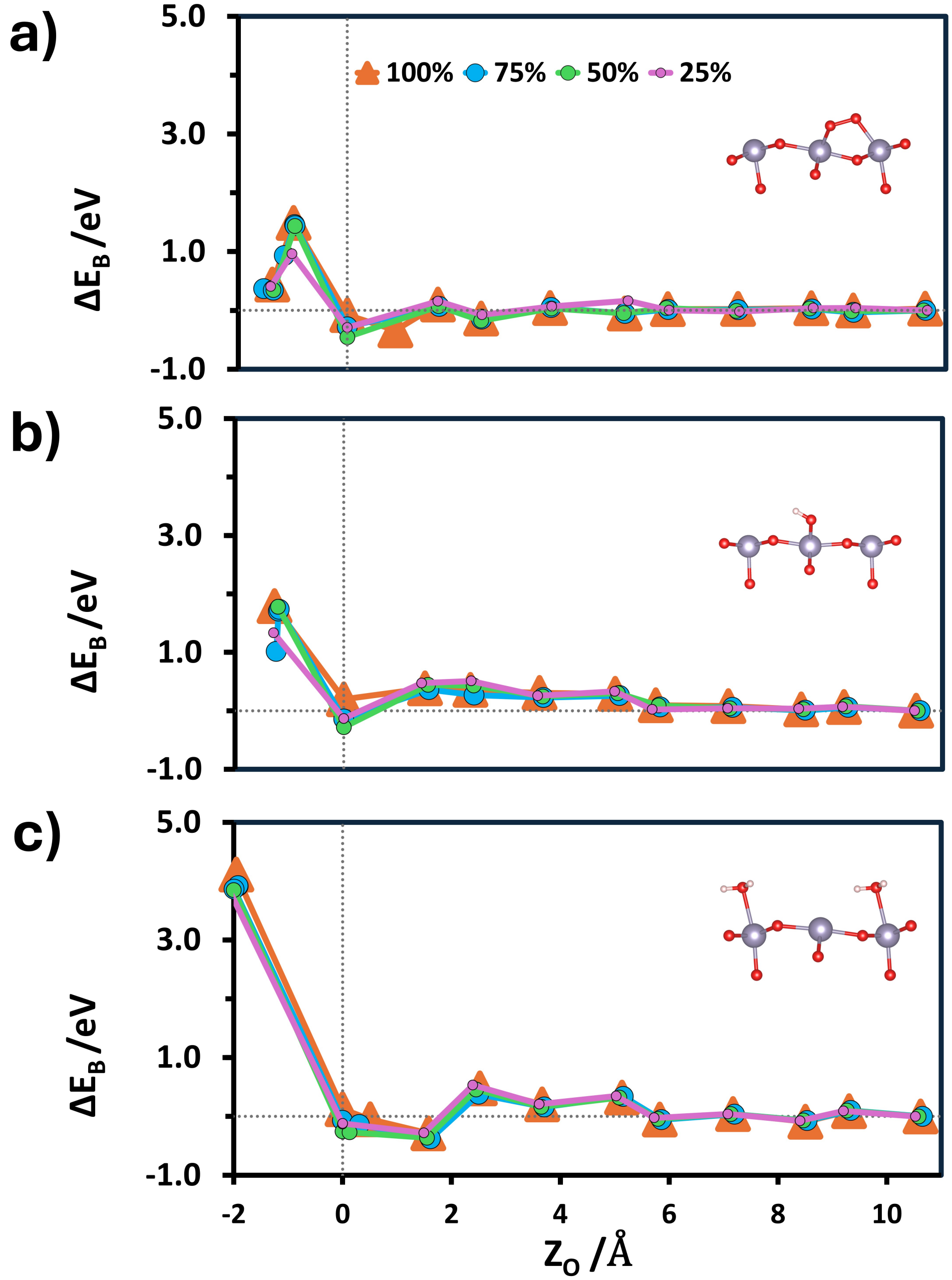

Figure 6: O 1s binding energies of adsorbates on fully reduced surfaces for intermediate coverages: O_2 (top panel), OH (middle panel), H_2O (bottom panel).

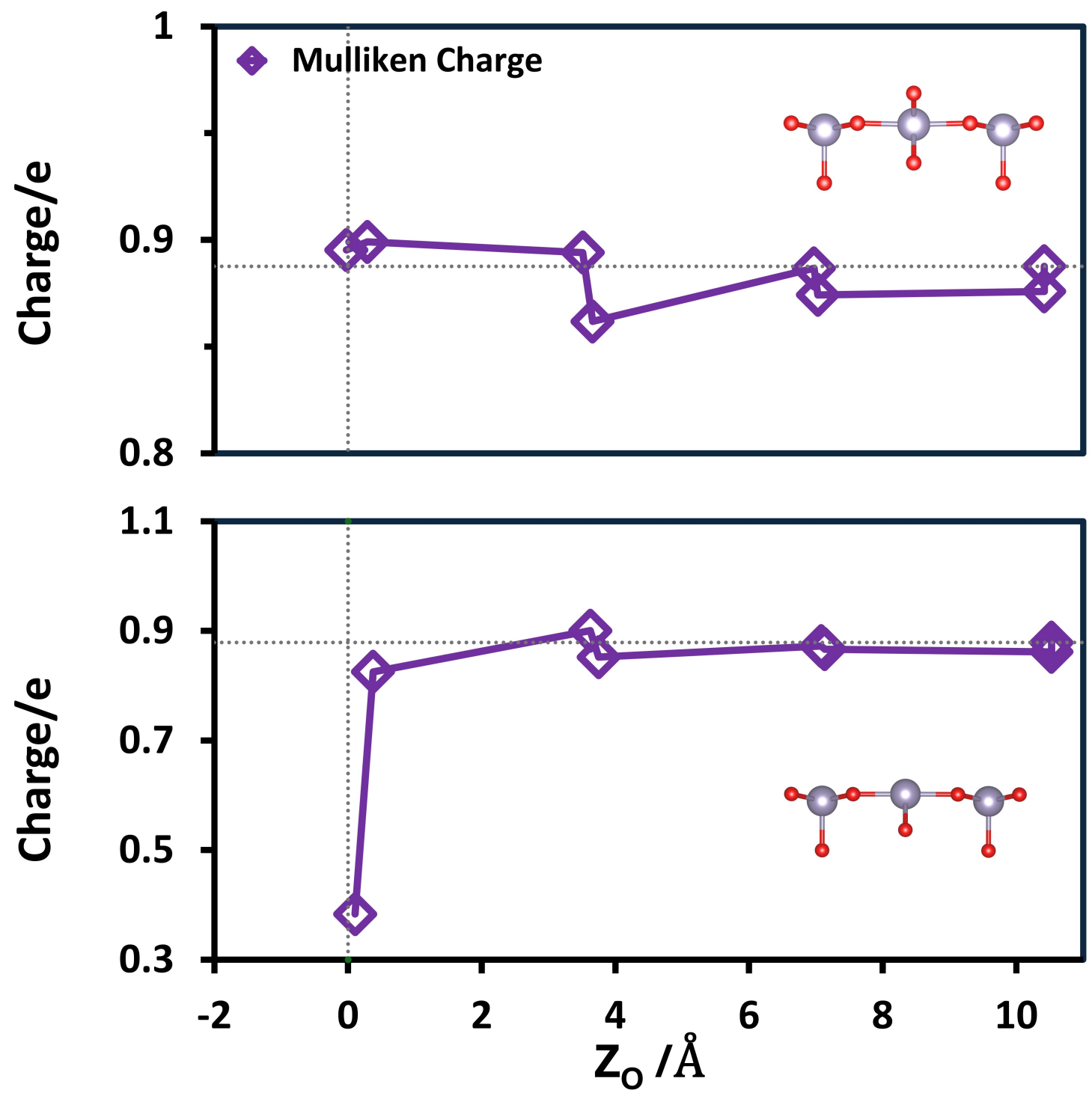

Figure 7: Comparison of the Mulliken charges of Sn atoms at the stoichiometric (top panel) and fully reduced (bottom panel) SnO_2 surfaces.
